# Triple gene mutations boost amylose and resistant starch content in rice: insights from *sbe2b*/*sbe1*/OE-*Wxa* mutants

**DOI:** 10.3389/fpls.2024.1452520

**Published:** 2024-08-14

**Authors:** Xiaoqiong Chen, Qiaoling Guo, Xiaoli Yang, Meng Yuan, Jianguo Song, Hongyan Fu, Hongyu Zhang, Peizhou Xu, Yongxiang Liao, Asif Ali, Kangxi Du, Xianjun Wu

**Affiliations:** State Key Laboratory of Crop Gene Exploration and Utilization in Southwest China, Rice Research Institute, Sichuan Agricultural University, Chengdu, China

**Keywords:** resistant starch, amylose, OE-Wx a, triple mutant, agronomic trait

## Abstract

Previous studies have modified rice's resistant starch (RS) content by mutating single and double genes. These mutations include knocking out or reducing the expression of *sbe1* or *sbe2b* genes, as well as overexpressing *Wx^a^*. However, the impact of triple mutant *sbe2b*/*sbe1*/OE-*Wx^a^* on RS contents remained unknown. Here, we constructed a double mutant with *sbe2b*/RNAi-*sbe1*, based on *IR36ae* with *sbe2b*, and a triple mutant with *sbe2b*/RNAi-*sbe1*/OE-*Wx^a^*, based on the double mutant. The results showed that the amylose and RS contents gradually increased with an increase in the number of mutated genes. The triple mutant exhibited the highest amylose and RS contents, with 41.92% and 4.63%, respectively, which were 2- and 5-fold higher than those of the wild type, which had 22.19% and 0.86%, respectively. All three mutants altered chain length and starch composition compared to the wild type. However, there was minimal difference observed among the mutants. The *Wx^a^* gene contributed to the improvement of 1000-grain weight and seed-setting rate, in addition to the highest amylose and RS contents. Thus, our study offers valuable insight for breeding rice cultivars with a higher RS content and yields.

## Introduction

Rice (*Oryza sativa* L.) is a staple food for a large proportion of the global population. Starch, the main component of rice grains, provides up to 40% of the caloric intake for those who rely on rice as a staple food (Awika et al., 2011). Most starch in cereal products is digested rapidly in the upper gastrointestinal tract; however, resistant starch (RS) is unaffected by enzymatic hydrolysis and hence escapes degradation in the stomach and small intestine (Englyst et al., 1982; Yang et al., 2016).

RS diets have numerous health benefits, such as reducing pathogenic infections, colon cancer rates, postprandial glycemia, and low-density lipoprotein (LDL) serum cholesterol levels (Zhang et al., 2015; Marlatt et al., 2018; Medina-Remón et al., 2018). A high-RS diet is particularly effective in reducing the risk of developing type-2 diabetes (Topping et al., 2003), a condition that is increasingly prevalent worldwide and affects many young individuals. Consequently, several studies have focused on enhancing RS content through genetic breeding, biotechnology techniques, and physical and chemical methods (Bird et al., 2008; Jiang et al., 2010; Ambigaipalan et al., 2014).

The RS content in rice is influenced by various factors, including amylose content, the ratio of amylose to amylopectin, and molecular characteristics of starch such as chain structure, granule size, and amorphous content. These characteristics are determined by genetic factors, such as genes involved in the starch biosynthesis pathway and transcription factors (Guzman et al., 2017; Bemiller, 2019). Starch biosynthesis in rice is a complex process involving different enzymes namely, Granule-bound starch synthase I (GBSSI), starch synthase (SS), disbranching enzyme (DBE), and starch branching enzyme (SBE). These enzymes play a role in the production of RS by affecting amylose content, chain length, granule size, gel consistency, and gelatinization temperature (Hennen-Bierwagen et al., 2008). GBSSI plays a crucial role in the amylose formation in the rice endosperm and is encoded by the *waxy* allele (*Wx*) gene on chromosome 6 (Liu et al., 2009). To date, nine *Wx* alleles have been identified: *Wx^a^*, *Wx^b^*, *Wx^in^*, *Wx^lv^*, *Wx^op^*, *Wx^mq^*, *Wx^mp^*, *Wx^la^*, and *Wx* (Zhou et al., 2021). Numerous studies have demonstrated that *Wx^a^* leads to high amylose content in *indica* rice varieties (Ayres et al., 1997; Wanchana et al., 2003; Mikami et al., 2008; Liu et al., 2009; Yang et al., 2013; Zhang et al., 2019). SSs catalyze the formation of linear α-1,4-linked glucan chains while DBEs and pullulanase are associated with semicrystalline amylopectin (Wattebled et al., 2005; Fujita et al., 2009). SBEs facilitate chain transfer by cleaving an α-1,4 linkage following the α-1,6 linkage condensation (Tanaka et al., 2004). Rice possesses three SBE isoforms: SBEI, SBEIIa, and SBEIIb (Zhu et al., 2012; Yang et al., 2016).

Considerable studies have shown that RS content is tightly related to amylose content, both of which are determined by genes involved in the starch biosynthesis pathway. It has been found that the downregulation of the *SBEII* gene can significantly increase amylose and RS content (Wei et al., 2010; Butardo et al., 2011; Zhao et al., 2015). Additionally, in *indica* rice, higher expression of *Wx^a^* has been shown to significantly increase amylose and RS contents (Zhou et al., 2016). Thus, these studies have focused on improving amylose and RS content by manipulating the expression of genes related to the starch biosynthesis pathway, for example, single mutants have been created to increase RS content by either knocking out or reducing *ss3a* or *sbe2b*, or by overexpressing *GBSSI*. Double mutants have also been utilized such as those with *ss3a* and *sbe2b*, *sbe1* and *sbe2b*, as well as overexpression of *Wx^a^* combined with knockout of *OsSBEIIb* (Shim et al., 2022). However, the effects of triple mutants involving *sbe2b*/RNAi-*sbe1*/OE-*Wx^a^* on amylose and RS contents remain unknown.

Our study aimed to determine the impact of mutations in three specific genes on RS formation to provide useful information for breeding cultivars with higher RS content. To achieve this, we created double and triple mutants, by knockdown of *SBEI* (Os06g0726400) expression in *IR36ae* with the *sbe2b* mutant, and overexpression of *Wx^a^* (Os06g0133000) in *IR36ae* and RNAi *SBEI*, respectively. Subsequently, we accessed the agronomic traits, measured amylose and RS contents, analyzed starch molecular characteristics, and examined the expression of genes involved in the starch biosynthesis pathway.

## Materials and methods

Wild-type IR36 was selected from the resources available at Sichuan Agricultural University, China. *IR36ae* (single mutant) was obtained from a mutant library of Sichuan Agricultural University. This mutant was induced using Ethyl methanesulfonate (EMS) as a mutagen, located at the splice site between exon and intron 11. This resulted in a change from G to A, causing a premature stop codon and producing a truncated form of BEIIb (Os02g0528200).

### Plasmid construction and transformation

To prepare the double mutant (*sbe2b*/RNAi-*sbe1*), we followed the same method as described by (Sallaud et al., 2003) to knock down the expression of *OsBEI* (Os06g0726400) based on *IR36ae* with the mutation of *sbe2b.* A 320-bp cDNA fragment belonging to a region encoding sequence in rice cultivar IR36 cDNA was amplified. The fragments were inserted on both sides of the intron in opposite orientations into the binary vector pTCK303. Afterward, it was cloned into the binary vector pCambia1300. To prepare the triple mutant (*sbe2b*/RNAi-*sbe1/OE-Wx^a^*), we overexpressed *Wx^a^* based on the double mutant according to the procedure described by (Luo et al., 2022). To summarize, we first amplified a full-length 1833-bp fragment from the cDNA sequence of rice cultivar IR36, next we cloned it into the binary vector pCambia1300. The transgenic plants were generated using Agrobacterium-mediated transformation and confirmed using gene-specific primers (**Supplementary Table 1**). These transgenic plants were observed for three consecutive years.

### DNA extraction

Fresh leaves were collected from each plant to extract genomic DNA using the CTAB method. PCR was performed by following these steps: initial denaturation at 95°C for 5 minutes, followed by 35 cycles of denaturation at 95°C for 30 seconds, annealing at 57°C for 30 seconds, extension at 72°C for 30 seconds, and a final extension at 72°C for 5 minutes.

### Real-time qPCR analysis

The spikelets were harvested on the 5th and 10th day of flowering. All spikelets samples were immediately frozen in liquid nitrogen and stored at -80°C until use. Total RNA was extracted from dehulled seeds using an RNA extraction reagent (Vazyme Co., Ltd, Nanjing, China) following the manufacturer's protocol. The extracts were treated with DNase I (Ambion). For cDNA synthesis, 2 μg of total RNA from caryopses was used with SuperScript III (Invitrogen). qPCR experiments were performed using gene-specific primers (**Supplementary Table 2**) in the SsoFast EvaGreen Supermix reaction system (Bio-Rad) on a CFX96 Real-Time System (Bio-Rad Co., Ltd, Singapore), according to the manufacturer's instructions. The rice actin gene (LOC_Os03g50885) was used as an internal reference.

### Sample preparation for amylose and RS contents and starch molecular characteristics

Wild type, single mutants (*IR36ae* with *sbe2b*), double mutants (*sbe2b*/RNAi-*sbe1*), and triple mutants (*sbe2b*/RNAi-*sbe1/OE-Wx^a^*) were planted in Wenjiang, Chengdu, China. Mature seeds were randomly selected from the wild-type and single, double, and triple mutants, with each group, replicated three times. All samples underwent milling refinement using SINOGRAIN, China Grain and Oil Reserve Co., Ltd. in Suzhou, China. The milled rice powder was then mixed and sifted through a 100-mesh sieve. These milled flours were used for further experiments.

### Determination of amylose and RS contents

The amylose contents of the milled rice flour samples were measured according to the method described by Tao et al. (2019). The RS content was examined using a Resistant Starch Assay Kit (Megazyme Co., Ltd. Bray, Ireland) and following the manufacturer's protocol which is based on the method described by Parween et al. (2020).

### Rapid visco analysis

The pasting properties of the milled rice were investigated using a rapid viscosity analyzer (Brabender Micro Visco-Amylo-Graph Co., Ltd. Brabender, Germany) and analyzed using Thermal Cycle for Windows software. Details of this procedure have been described (Zhang et al., 2013).

### Starch isolation

Starch samples from each treatment group were isolated following the method described by Lu and Lu (2012), with minor modifications. Briefly, samples (20 g) were soaked in 100 mL of ultrapure water containing sodium metabisulfite and 10 mg/g alkaline protease at 42°C for 24 h. The samples were mixed in a blender and sifted through a 200-mesh sieve. The resulting slurry was collected and allowed to stand for 12 hours. This step was repeated 5–8 times until the settled starch layer was purified. Starch samples were collected and air-dried. Samples were stored at 20°C until further use. Starch isolation was employed to analyze various properties including swelling power, water solubility, chain length, molecular weight, branching degree, granularity distribution, and granule morphology.

### Swelling power and water solubility determination of starch

The swelling power and water solubility of starch were measured at 95°C for 20 min following the method described by De la hera et al. (2013).

### Determination of starch granule morphology

To examine the endosperm cross-section, rice seeds were fractured along the short axis and fully dried under low pressure. The surfaces were sputter-coated with gold and observed under a scanning electron microscope (Zeiss, Oberkochen, Germany).

The starch granule morphology was observed following the protocol described by Zou et al. (2020). Samples (100 mg) were mounted on a metal stub, covered with gold, and then observed and photographed using a Zeiss Merlin Compact scanning electron microscope (SEM, Zeiss, Oberkochen, Germany).

### Determination of granule size

To a 100 mg sample in EP tubes, 1 mL of 75% alcohol was added and mixed using ultrasound. Samples were analyzed using a Mastersizer 3000 laser diffraction particle size analyzer (Malvern Instruments Ltd., Worcestershire, UK). All samples were measured in triplicate.

### Starch molecular weight determination

Starch (5 mg) was thoroughly mixed with 5 mL of 0.5% LiBr (w/w) in DMSO solution and heated in a thermomixer at 80°C for 3 h, as described by Zou et al. (2020). The molecular weight of the starch was determined using the gel permeation chromatography-refractive index-multiangle laser light scattering (GPC–RI–MALLS) method with an Optilab T-rEX differential refractive index detector (Wyatt Technology) and a DAWN HELEOSII (Wyatt Technology). Data were analyzed using ASTRA v6.1 (Wyatt, Santa Barbara, CA, USA). Three biological replicates were used for each sample.

### Determination of branch chain length distribution

The branch chain length distribution of amylopectin was measured using an ICS-5000 high-performance anion-exchange chromatograph (Thermo Fisher Scientific, Waltham, MA, USA) with a DionexTM CarbopacTM PA10 anion-exchange column, as described by Li et al. (2018). Three biological replicates were used for each sample.

### Analysis of X-ray diffraction patterns and branching degrees

To obtain XRD patterns, starch samples (100 mg each) were scanned using an X'Pert Pro X-ray diffractometer (PANalytical, Almelo, Netherlands) and was performed by Sanshu Biotechnology Co., LTD (Shanghai, China). The X-ray source used was Cu K_α_ with 0.154-nm filtered radiation. Each 100-mg sample was scanned at scattering angles of 5–60° (2θ) using a scanning rate of 4°/min. The degree of crystallinity was calculated using MDI-Jade v5.0 (Material Data, Inc., Livermore, CA, USA). A BioSpin NMR spectrometer (Bruker) was used to determine the branching degree. Three biological replicates were used for each sample.

### Statistical analysis

Mean values and standard deviations were calculated for each sample in each measurement. One-way analysis of variance (ANOVA) and Pearson correlation analysis was conducted using SPSS version 18.0 (SPSS, Inc., Chicago, IL, USA). Significant differences were assessed with Student's *t*-test and considered statistically significant at *p* < 0.05.

## Results

### Mutant construction and examination of expression levels of genes involved in the starch biosynthesis pathway

The *IR36ae* mutant with *sbe2b* was identified by sequencing (**Supplementary Figure 1A**). Double mutants of *sbe2b*/RNAi-*sbe1* and triple mutants of *sbe2b*/RNAi-*sbe1/*OE*-Wx^a^* were successfully generated. RNAi-*sbe1* and OE-*Wx^a^* were identified through gel band analysis (**Supplementary Figures 1B, C**).

**Figure 1:**
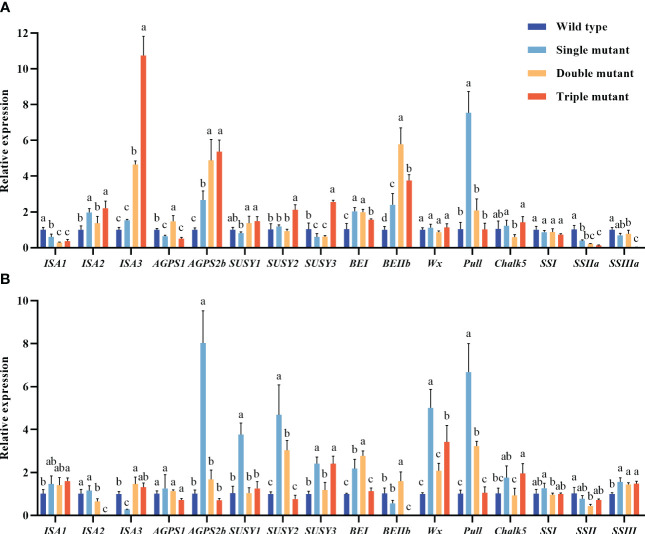
Genes expression levels in caryopsis from starch biosynthesis pathway. **(A)** Genes expression from 5 days after flowering; **(B)** Genes expression from 10 days after flowering. Values are expressed as mean ± SD (n=3). Single mutant represents mutation of gene *sbe2b*; Double mutant represents mutation of genes *sbe2b* and RNAi-*sbe1*; Triple mutant represents mutation of genes *sbe2b*, RNAi-*sbe1* and over expressed gene *Wx^a^*. Different superscript letters represent significant differences at *P* < 0.05, and significant was compared in same index.

First, we assessed important genes involved in starch biosynthesis to determine the impact of *SBEIIB*, *SBEI*, and *Wx^a^* on expression levels of other genes related to the starch biosynthesis pathway, subsequently affecting amylose and RS levels (**Figure 1**). The expression levels of *ISA3* (after 5 days of flowering) in the double and triple mutants were 5 and 11 times higher, respectively, compared to the wild type. The study further revealed that the lack of activity of *SBEIIb* and suppressed expression of *SBEI* had a significant impact on the expression of *SSIIa* and *SSIIIa*. However, they have a significant increase in the expression of *isoamylase 3* (*ISA3*), *ADP-glucose pyrophosphorylase 2b* (*AGPSIIb*), and *Wx^a^*. The expression levels of *Wx^a^* and *pullulanase* after 10 days of flowering in single mutants were more than 5 and 9 times higher, respectively, as compared to the wild type. This indicates that mutated *sbe2b* and RNAi*sbe1* influence the expression of the other genes associated with the starch biosynthesis pathway.

### Amylose and RS contents were significantly increased with an increase in the number of mutated genes

**Figure 2:**
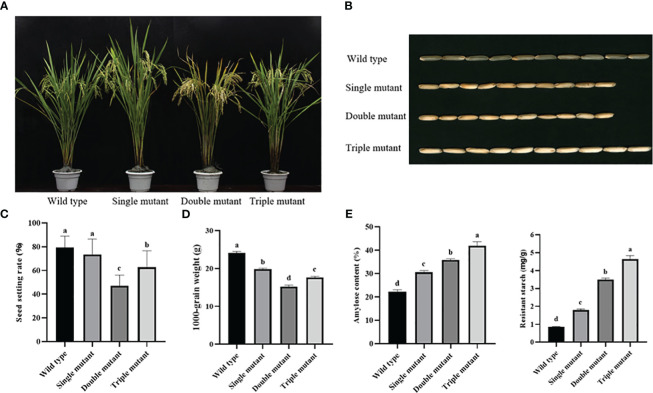
Phenotype, seed retting rate, 1000-grain weight, amylose and resistant starch content in wild type and three mutants. **(A)** Phenotype of plant; **(B)** Appearance of mature dehulled grains; **(C)** Seed setting rate; **(D)** 1000-grain weight; **(E)** Amylose content; **(F)** Resistant starch content. All agronomic traits and data were obtained from 2022. Single mutant represents mutation of gene *sbe2b*; Double mutant represents mutation of genes *sbe2b* and RNAi-*sbe1*; Triple mutant represents mutation of genes *sbe2b*, RNAi-*sbe1* and over expressed gene *Wx^a^*. Values are means ± SD (n=3). Different superscript letters represent significant differences at *P* < 0.05.

We recorded agronomic traits for three consecutive years because amylose content is influenced by various factors, including agronomic traits. The results exhibited that the wild type had the highest seed-setting rate at 79.51%, followed by single and triple mutants at 73.45% and 62.67% respectively. The lowest seed setting was observed for a double mutant with a 46.96% value. The 1000-grain weight was similar to the seed setting rate, with the highest weight observed in wild type, followed by single mutant, and triple mutant, and the lowest was in double mutant, with weights of 24.18, 19.80, 17.61, and 15.18 g, respectively. In brief, the double mutant had a notable impact on seed setting rate and 1000-grain weight, resulting in a significant decrease in the yield (**Figures 2A–D** and **Supplementary Table 3**). In contrast, the triple mutant significantly restored the 1000-grain weight and seed-setting rate similar to those of the single mutants. This indicates that OE-*Wx^a^* contributes to the restoration of yield.

The amylose and RS contents were analyzed for three consecutive years. Their range was highest in the triple mutant over three years, followed by the double and single mutants, with the lowest contents in the wild type (**Figures 2E, F** and **Supplementary Table 4**). In 2022, the triple mutant had amylose and RS contents of 41.92% and 4.63%, respectively. The double mutants had lower contents with 35.87% for amylose and 3.49% for RS. The single mutant has even lower contents of amylose (30.57%) and RS (1.79%). The lowest contents were found in the wild-type, at 22.19% and 0.86% for amylose and RS, respectively. This indicates that amylose and RS content significantly increased as the number of mutated genes increased.

### RVA properties gradually decreased with an increasing number of mutated genes

**Figure 3:**
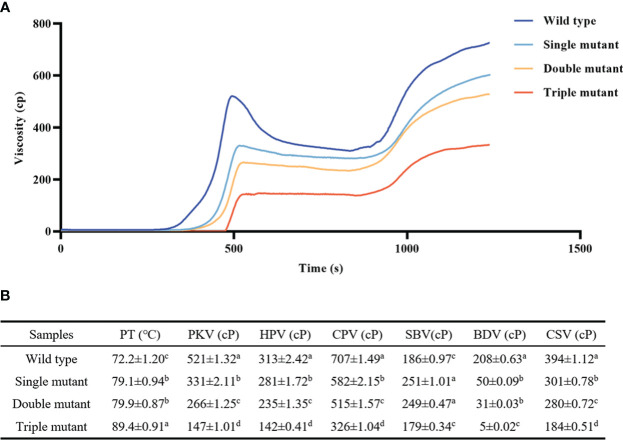
Rapid viscosity analyzer (RVA) spectra of samples from wild and three mutants. **(A)** RVA spectral; **(B)** RVA spectral indexes. PT, peak temperature; PKV, peak viscosity; HPV, hot paste viscosity; CPV, cool paste viscosity; SBV, setback viscosity; BDV, breakdown viscosity; CSV, consistence viscosity. Single mutant represents mutation of gene *sbe2b*; Double mutant represents mutation of genes *sbe2b* and RNAi-*sbe1*; Triple mutant represents mutation of genes *sbe2b*, RNAi-*sbe1* and over expressed gene *Wx^a^*. Values are expressed as the mean ± standard deviation (n = 3). Superscript letters represent significant differences at *p* < 0.05, and significant was compared in same index.

The study then examined RVA properties to identify whether *Wx^a^*, *sbe2b*, and *sbe1* have an impact on these properties. Analysis of RVA spectra showed that most of the indices showed a decreasing tendency, except for peak temperature (PT). For instance, the breakdown viscosity in the wild type was 4, 7, and 41 times higher than that of single, double, and triple mutants, respectively (**Figure 3**). This indicates that *sbe1*, *sbe2b*, and *Wx^a^* all significantly influenced the RVA properties, with their effect gradually diminishing with an increasing number of mutated genes.

### Genes *SBEIIb* and *Wx^a^* influenced swelling power

The gel consistency was the highest in the triple mutant (3.03 cm), followed by the wild type (2.77 cm), double mutant (2.73 cm), and single mutant (2.60 cm) (**Table 1**). Water solubility was highest in the double mutant (8.04%), followed by the single mutant (6.99%), triple mutant (6.66%), and wild type (5.92%). However, the swelling power showed a different pattern compared to gel consistency and water solubility. It was highest in the wild type (12.56 g/g), followed by the double mutant (8.75 g/g), single mutant (8.67 g/g), and triple mutant (7.69 g/g). This indicates that the *SBEIIb* and *Wx^a^ genes* significantly influence swelling power.

**Table 1:** GC, water solubility and swelling power of wild type and three mutants.

Samples	GC (cm)	Water solubility (%)	Swelling power (g/g)
Wild type	2.77 ± 0.06^b^	5.92 ± 0.20^b^	12.56 ± 0.31^a^
Single mutant	2.60 ± 0.10^c^	6.99 ± 0.33^ab^	8.67 ± 0.33^b^
Double mutant	2.73 ± 0.25^b^	8.04 ± 1.36^a^	8.75 ± 0.18^b^
Triple mutant	3.03 ± 0.06^a^	6.66 ± 0.71^ab^	7.69 ± 0.63^c^

GC: gel consistency. Single mutant represents mutation of gene sbe2b; Double mutant represents mutation of genes sbe2b and RNAi-sbe1; Triple mutant represents mutation of genes sbe2b, RNAi-sbe1 and over expressed gene Wx^a^. Values are expressed as mean ± SD (n=3). Different superscript letters represent significant differences at P < 0.05, and significant was compared in same index.

### Genes *sbe1*, *sbe2b*, and OE-*Wx^a^* altered the starch granule morphology

**Figure 4:**
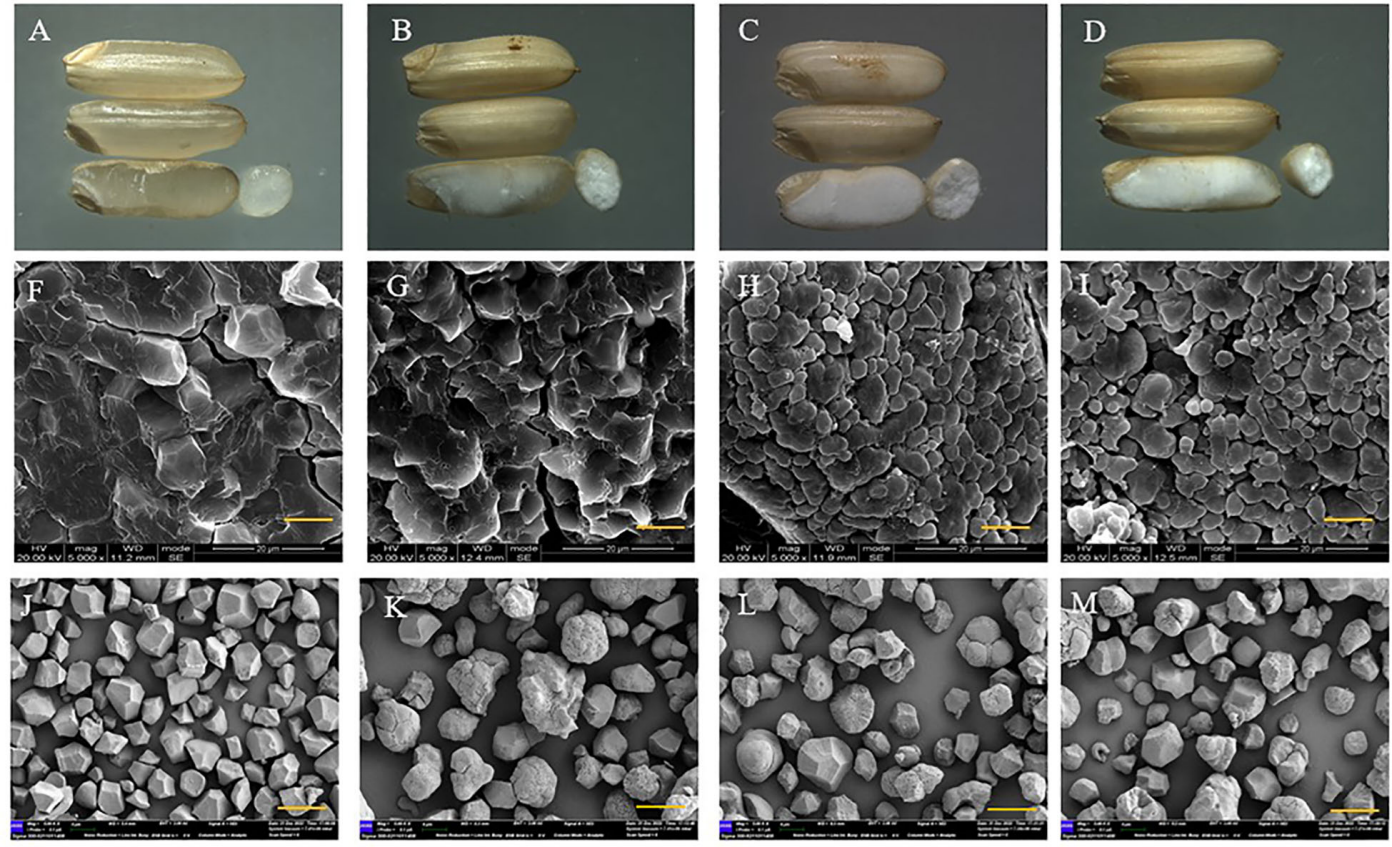
Morphology of seeds and endosperm from wild type and three mutants. **(A–D)** Morphology of seeds for wild type, single mutant, double mutant, and triple mutant, respectively; **(F–I)** Scanning electron micrographs of cross section of grains at 2000 times for wild type, single mutant, double mutant, and triple mutant, respectively, bar represses 20 μm; **(J–M)** Scanning electron micrographs of starch granules at 7000 times for wild type, single mutant, double mutant, and triple mutant, respectively, bar represses 3 μm. Single mutant represents mutation of gene *sbe2b*; Double mutant represents mutation of genes *sbe2b* and RNAi-*sbe1*; Triple mutant represents mutation of genes *sbe2b*, RNAi-*sbe1* and over expressed gene *Wx^a^*.

**Figure 5:**
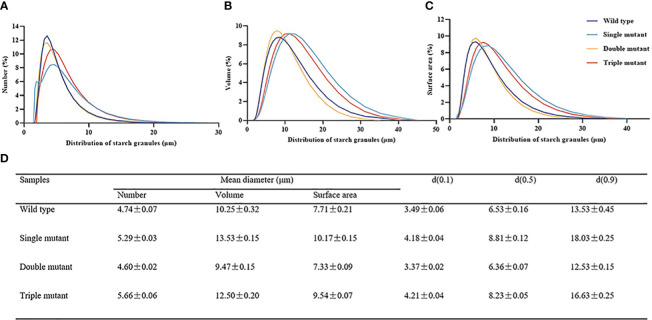
Particle size of starch extracted from the grains of wild type, single, double and triple mutant. **(A–C)**, Distributions of **(A)** size, **(B)** volume, and **(C)** surface area among starch granules. **(D)** Distribution of the granule diameter of wild type, single mutant, double mutant and triple mutant. Single mutant represents mutation of gene *sbe2b*; Double mutant represents mutation of genes *sbe2b* and RNAi-*sbe1*; Triple mutant represents mutation of genes *sbe2b*, RNAi-*sbe1* and over expressed gene *Wx^a^*. Values are expressed as the mean ± standard deviation (n = 3).

First, we observed a gradual decrease in transparency with an increasing number of mutated genes. The triple mutant indicated the lowest transparency, while the wild type displayed high transparency (**Figures 4A–D**). The starch granule morphology was determined by examining the cross-section, and the results revealed that the granule morphology of the wild type was crystalline and closely spaced, while the single, double, and triple mutants had rounder and looser granules (**Figures 4F–I**). Granule morphology was further observed using isolated starch granules, which confirmed that the crystalline structure was only present in the wild type, while all three mutants displayed rounder and looser granules (**Figures 4J–M**). We also examined the particle size of starch, and the results were in the following order: single mutant > triple mutant > wild type > double mutant. It is indicated that the single mutant had the largest starch particles. The triple mutant had a significantly larger starch particle size than the wild-type and smaller particles compared to the single mutant. In addition, there was no significant difference in starch particle size between the wild type and double mutants (**Figure 5**). These findings indicate that genes *SBE1*, *SBEIIb*, and *Wx^a^* all play a role in regulating starch granule morphology.

### *sbe1, sbe2b* and *Wx^a^* genes altered polydispersity, but no cumulative effect was observed with an increasing number of genes

Polydispersity is a measure of the broadness of molecular weight distribution, and can be calculated via X-ray-based analysis (Zhang et al., 2020). The single mutant significantly decreased both the number average molecular weight (Mn) and mean molecular weight (Mz). These values were more than twice as low as that of the wild type. The Mn and Mz values of the double and triple mutants were identical to those of the single mutant. Next, we analyzed the two most important polydispersity indices: Mw/Mn and Mz/Mn. These indices were significantly lower in the wild type compared to the single, double, and triple mutants. However, no significant differences were observed among the three mutants (**Table 2**). This indicates that genes *SBE1*, *SBEIIb*, and *Wx^a^* all influence polydispersity, but their effects are not cumulative.

**Table 2:** Starch molecular weights of wild and three mutants.

Samples	Mn (KDa)	Mp (KDa)	Mw (KDa)	Mz (KDa)	Mw/Mn	Mz/Mn
Wild type	39299.97 ± 69.35^a^	106545.21 ± 132.74^a^	80453.95 ± 57.41^a^	190951.48 ± 398.55^a^	2.05 ± 0.03^b^	4.86 ± 0.24^c^
Single mutant	17908.95 ± 21.72^c^	90005.19 ± 78.93^b^	47417.56 ± 36.84^c^	145402.12 ± 187.51^b^	2.65 ± 0.04^a^	8.12 ± 0.43^a^
Double mutant	23355.18 ± 25.20^b^	95515.94 ± 91.85^b^	59412.73 ± 42.68^b^	159032.01 ± 254.99^b^	2.54 ± 0.03^a^	6.81 ± 0.30^b^
Triple mutant	20245.19 ± 19.87^b^	88479.58 ± 66.42^c^	50485.92 ± 64.27^b^	137864.39 ± 226.37^c^	2.49 ± 0.02^a^	6.81 ± 0.35^b^

Single mutant represents mutation of gene sbe2b; Double mutant represents mutation of genes sbe2b and RNAi-sbe1; Triple mutant represents mutation of genes sbe2b, RNAi-sbe1 and over expressed gene Wx^a^. Mn: number average molecular weight; MW: weight average molecular weight; MZ: mean molecular weight; MP: peak molecular weight. Mw/Mn and MZ/Mn: polydispersity Index. Values are expressed as mean ± SD (n=3), and significant was compared in same index.

### All three genes altered chain length and starch composition, but no cumulative effect was observed with an increasing number of genes

**Figure 6:**
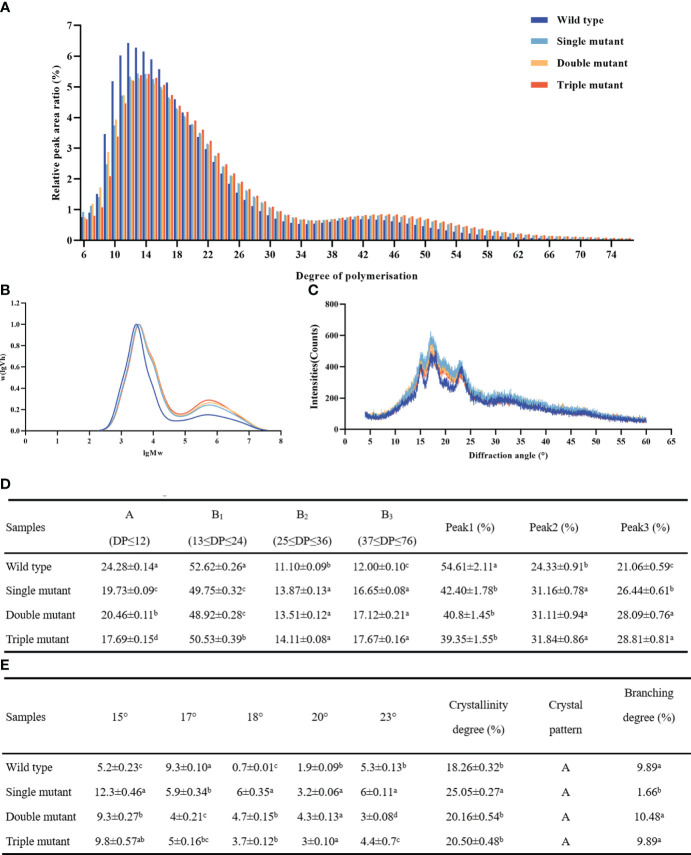
Starch chain length and X-ray diffraction (XRD) patterns of starch granules in grains collected from wild type and three mutants. **(A)** Detection of chain lengths from six to 76 degrees of polymerization (DP) via ion chromatography. **(B)** Detection of chain lengths with ≥ 100 DP in via gel permeation chromatography. **(C)** XRD patterns. Peak 1, A-chains and short B-chains of amylopectin; peak 2, intermediate and long chains of amylopectin; peak 3, amylose with chain length and DP ≥ 100. **(D)** Different chain length distribution; **(E)** Crystallinity degree, crystal pattern and branching degree. Single mutant represents mutation of gene *sbe2b*; Double mutant represents mutation of genes *sbe2b* and RNAi-*sbe1*; Triple mutant represents mutation of genes *sbe2b*, RNAi-*sbe1* and over expressed gene *Wx^a^*. Values are expressed as the mean ± standard deviation (n = 3). Superscript letters represent significant differences at *p* < 0.05.

*SBEIIb* plays a specific role in the formation of branches in the crystalline lamellae of amylopectin clusters in the rice endosperm and affects the distribution of chain length in amylopectin. The current study revealed, that the single mutant *sbe2b* significantly decreased A (DP ≤ 12) and B1 (13 ≤ DP ≤ 24) chains, these chains were 19% and 6% lower, respectively, compared to the wild type. On the other hand, *Sbe2b* significantly increased B2 (25 ≤ DP ≤ 36), B3 (37 ≤ DP ≤ 76), peak 2, and peak 3 (DP ≥ 100) by 25%, 37%, 28%, and 26%, respectively, compared to the wild type (**Figures 6A, B, D**). Thus, all three genes altered chain length and starch composition, but there was no cumulative effect when more genes were involved.

### The *sbe1, sbe2b*, and OE-*Wx^a^* genes did not alter the diffraction patterns of starch

The highly ordered crystalline structure of starch is attributed to intra- and intermolecular hydrogen bonding. XRD is a widely used method to assess this structure (Garg and Jana, 2011). Our study showed that single, double, and triple mutants displayed A-type diffraction patterns similar to the wild type (**Figure 6C**). However, we found that peak degrees significantly differed between the wild type and mutants as well as among the mutants. We further found that the 17° peak was highest in the wild type, followed by the single and triple mutants. In contrast, the single mutant had the highest peak (18°), followed by the double and triple mutants, while the wild type had the lowest intensity (**Figure 6E**). In brief, mutated *sbe1*, *sbe2b*, and OE-*Wx^a^* genes did not alter the diffraction patterns of starch.

### The *sbe1, sbe2b*, and *Wx^a^* genes significantly influenced crystallinity and branching degree

The single mutant displayed the highest degree of crystallinity (25.05%), while the wild type had the lowest value (18.26%). In addition, the degree of crystallinity did not significantly differ between the double (20.16%) and triple mutants (20.50%). Our study further revealed that no notable variation was observed among the wild-type, double-mutant, and triple-mutant strains. Regarding the branching degree, the lowest value was observed in the single mutant (1.66%), but there were minimal differences among the wild-type, double, and triple mutants, similar to the degree of crystallinity (**Figure 6E**). This indicates that *sbe2b* greatly influenced the branching degree, while *sbe1* contributed in restoring the reduced branching degree caused by the mutation of *sbe1*.

## Discussion

A daily diet rich in RS has been proven beneficial to human health, particularly in diabetic people (Topping et al., 2003). However, the previous study indicated that increasing RS content led to decreased yield due to lower seed weight. Thus, it is interesting to provide information to breed rice cultivars with greater RS content and higher yield.

### The *sbe1, sbe2b*, and *Wx^a^* genes significantly influenced the expression of genes related to starch biosynthesis pathway

We first, evaluated the key genes related to starch biosynthesis to determine the impact of *SBEIIB*, *SBEI*, and *Wx^a^* on the expression levels of other genes, as well as on amylose and RS levels (**Figure 1**). The results indicated that the absence of *SBEIIb* activity and suppressed expression of *SBEI* significantly inhibited the expression of *SSIIa* and *SSIIIa*, while significantly increased the expression of *isoamylase 3* (*ISA3*), *ADP-glucose pyrophosphorylase 2 b* (*AGPSIIb*), and *Wx^a^*. However, our findings contradict those of Baysal et al. (2020) who reported that lack of SBEIIb activity triggered the activity of SSI, SSIIa, and AGPSIIb, while leading to downregulation of the genes encoding GBSS, SDBE, pullulanase, and starch phosphorylase. Because of these conflicting results, future studies should examine amylose and RS contents combined with the gene expression levels, to identify genes contributing to the formation of higher RS content. This would aid in providing information for breeding rice cultivars with a higher RS content.

### OE-*Wx^a^* significantly increased RS content and restored agronomic traits

Our study focused on observing agronomic traits, which are directly related to starch contents. The results indicated that the double mutant had a significant decrease in the seed setting rate and 1000-grain weight compared to the single mutant, ultimately leading to a significant decrease in yield (**Figures 2A–D** and **Supplementary Table 3**). In contrast, the triple mutant significantly restored the 1000-grain weight and seed-setting rate to levels similar to those of the single mutants. Miura et al. (2021) reported that mutated *sbe1* did not affect seed weight, while the mutated *sbe2b* gene did have a significant impact on seed weight. They found that *sbe1* in the double mutant (*sbe1/sbe2b)* was able to restore the lower weight of seeds caused by lack of SBEIIb activity. However, our results were inconsistent with those of Miura et al., because the 1000-grain weight in the double mutant was 4.62 g lower than that in the single mutant. Therefore, our study not only achieved the highest RS and amylose contents but also found better agronomic traits. However, further studies are required to understand how *sbe1* and *sbe2b* regulate these traits.

The amylose and RS contents were measured, and the results showed that the triple mutant had the highest levels. We further found that induction of OE-*Wx^a^* based on *sbe2b*/RNAi*sbe1* increased 16.9% and 32.7%, 37.1% and 158.7%, 88.9% and 438.4%, respectively, when compared to the double mutant *sbe2b*/RNAi*sbe1*, single mutant with *sbe2b*, and wild type. Similar to our study, previous studies have reported that downregulation of *SBEII* can result in high amylose content in mutant rice, maize, wheat, and barley (Wei et al., 2010; Butardo et al., 2011; Zhao et al., 2015). Double mutant *sbe1*/*sbe2b* exhibited significantly increased amylose and RS contents compared to the single mutant *sbe2b* (Miura et al., 2021). Higher expression of *Wx^a^* significantly increased amylose and RS contents in *indica* rice compared to the wild-type (Zhou et al., 2016). Therefore, the triple mutant presented the highest RS content, by introducing OE-*Wx^a^*, and further enhancing amylose biosynthesis through repression of *sbe1* and *sbe2b* gene expression. In addition, Zhou et al. (2016) reported that defective *SSIIIa* leads to increased RS levels, which depend on the higher expression of *Wx^a^*. This indicates that mutations in the genes related to starch biosynthesis can further elevate RS content.

### The elevated RS content was related to altering physiochemical properties and starch molecular characteristics by mutated genes *sbe1, sbe2b,* and OE-*Wx^a^*

Our results showed that RS content gradually increased as the number of mutated genes increased. Previous studies showed that starch molecular characteristics play a significant role in RS formation (Zhou et al., 2018; Tao et al., 2022; Sangwongchai et al., 2023). Thus, our study needed to explore whether genes *sbe1*, *sbe2b*, and *Wx* play a role in RVA properties, swelling power, starch granule size, and molecular characteristics, ultimately affecting RS formation.

### The *sbe1, sbe2b*, and OE-*Wx^a^* all decreased RVA properties

The RVA properties which are vital taste values were checked, and the results showed that most of the indices exhibited decreasing tendencies, except for peak temperature (PT). For instance, the breakdown viscosity in the wild type was 4, 7, and 41 times higher compared to the single, double, and triple mutants, respectively (**Figure 3**). This indicates that *sbe1*, *sbe2b*, and *Wx^a^* all had a significant impact on the RVA properties. A study by Shim et al. (2022) reported that GBSSI combined with a *sbe2b* mutant that had a mutation in one T/C SNP in the 16^th^ exon, resulting in a change from leucine to proline, led to alteration in RVA and other physiochemical properties. Miura et al. (2022) reported that the high expression of *Wx^a^* based on *sbe2b* mutant had a significant affected on the thermal properties of starch, which aligns with the findings of our study. This indicates that *sbe1*, *sbe2b*, and *Wx^a^* impact RS and amylose content, along with altered physiochemical properties.

### The elevated RS content was related to decreasing swelling power by mutated genes *sbe2b* and OE-*Wx^a^*

Teng et al. (2016) reported that *Wx* alleles have an impact on the swelling property of starch. In our study, we observed that both *sbe2b* and *Wx* may decrease swelling power. It was evident from the triple mutant having the lowest swelling power, and the single mutant showing significantly lower swelling power than that of the wild type. However, there was no significant difference between the single and double mutants. Given the RS content, the results suggest that high RS content is associated with decreased swelling power due to the lack of SBEIIb and OE-*Wx* activity.

### The elevated RS content was related to altering starch molecular characteristics by mutated *sbe1*, *sbe2b*, and OE-*Wx^a^*

Butardo et al. (2011) discovered that *sbe2b* mutants, through the use of artificial microRNA- and hairpin RNA-mediated RNA silencing, altered the granule morphology of starch, resulting in rounder, looser, and more pronounced granules with larger spaces than that of the wild type. Similar to these results, Yang et al. (2016) also reported altered granule morphology. We further validated that the mutation of genes involved in starch biosynthesis leads to alterations in the starch granule structure and affects transparency. It was evident from observed differences in the granule morphology between the wild type and the single, double and triple mutants. The wild type displayed a crystalline and closely spaced, whereas the mutant presented rounder and looser granules (**Figures 4F–I**). The particle size of the starch also was examined, and the results showed that the single mutant had the largest starch particles. Interestingly, the starch particle size of the triple mutant was significantly larger compared to the wild type, while it was smaller compared to the single mutant. In addition, there was no significant difference in starch particle size between the wild type and double mutants (**Figure 5**). This indicates that the starch granule morphology is related to *SBE1*, *SBEIIb* and *Wx^a^* genes. By considering the RS contents, it can be deduced that the increased RS content is likely influenced by factors other than altering starch granule size caused by the mutated genes *sbe2b*, *sbe1*, and OE-*Wx^a^*.

The molecular weight was checked, and the results showed that the single mutant significantly decreased the number average molecular weight (Mn) and mean molecular weight (Mz), compared to the wild type. The Mn and Mz of the double and triple mutants were identical to those of the single mutant. Next, Mw/Mn and Mz/Mn were also analyzed and it was demonstrated that these indices were significantly lower in the wild type compared to the single, double, and triple mutants. No significant differences were observed among the three mutants (**Table 2**). This indicates that genes *SBE1*, *SBEIIb*, and *Wx^a^* all have an impact on polydispersity, but do not have cumulative effects. Combining RS content indicates that the molecular weight of starch is closely related to increased RS content due to mutated genes *sbe2b*, *sbe1*, and OE-*Wx^a^*.

Yang et al. (2016) found that the proportion of short chains (DP ≤ 13) in *sbe2b* with a single amino acid mutation was significantly lower compared to the wild type. On the other hand, there was a notable increase in long chains (DP ≥ 15). Unlike the report of Yang et al. (2016), our study showed that *sbe2b* significantly decreased A (DP ≤ 12) and B1 (13 ≤ DP ≤ 24) chains, which were 19% and 6% lower in the single mutant compared to the wild type, respectively. *Sbe2b* significantly increased B2 (25 ≤ DP ≤ 36), B3 (37 ≤ DP ≤ 76), peak 2, and peak 3 (DP ≥ 100) by 25%, 37%, 28%, and 26%, respectively, compared to those of the wild type (**Figures 6A, B, D**). Similar to our study, Shim et al. (2022) reported that A and B1 chains in *sbe2b* due to mutation in the 16^th^ exon, resulting in a change from leucine to proline, were significantly decreased compared to wild type. Conversely, B_2_, B3, and peak 3 (amylose) showed a considerable increase. Ying et al. (2022) reported that *IR36ae* which lacks the activity of *sbe2b* exhibited changes in the chain length, consistent with the study of Shim et al. We hypothesize that different mutant locations of *SBEIIb* result in distinct branched structures, impacting the formation of RS and the physicochemical properties of starch. Furthermore, by analyzing the expression levels of *SBEI* and *Wx^a^* in mutant with *sbe2b*, we concluded that the absence of SBEIIb activity leads to the reduction of A and B1 chains but an increase in the B2 and B3 chains due to the significantly increased activity of GBSSI. Thus, our study re-confirms that SBEIIb is responsible for synthesizing B_1_ chains rather than B2 or B3 chains. We observed that there was no cumulative effect on the ratio of B2, B3, peak 2, and peak 3, or the ratio of A, B1, and peak 1 as the number of mutated genes increased. This was because there were minimal differences among the various mutants (**Figure 6D**). Therefore, we discovered that the RS content gradually increased with the number of mutated genes, mainly due to the rise in amylose content.

The crystalline structure of starch was also examined. The results showed that the single, double, and triple mutants displayed A-type diffraction patterns similar to those of the wild type (**Figure 6C**). However, there were significant differences in the peak degrees between the wild type and mutants and also among the mutants as well. Particularly we observed that the 17° peak was highest in the wild type, followed by the single and triple mutants. On the other hand, at 18°, the peak was highest in the single mutant, followed by the double and triple mutants, and lowest in the wild type (**Figure 6E**). In a study by Yang et al. (2016), it was reported that the *sbe2b* mutant, with a single amino acid mutation, exhibited a C-type diffraction pattern, while the wild type displayed an A-type pattern. They also reported a B-type pattern due to a deficiency in BEIIB (Tappiban et al., 2022). Another study by Miura et al. (2021) reported that the *sbe1* single mutant displayed an A-type pattern, whereas the *sbe2b* single and *sbe2b*/*sbe1* double mutants displayed a B-type pattern. Consistent with our findings, Butardo et al. (2011) found that the *sbe2b* mutant, silenced using hairpin RNA-mediated RNA silencing, did not alter the diffraction patterns of starch in rice. We hypothesize that these different diffraction patterns are the results of mutations occurring in different locations of the genes, which impact the enzyme activity and subsequently lead to significant differences in starch structures.

Previous studies have reported that crystallinity and branching degree of starch influence RS formation (Jane et al., 1997). In the current study, we observed that the *sbe2b* significantly increased the degree of crystallinity, as the single mutant exhibited the highest degree of crystallinity. However, there was no significant difference in crystallinity, which did not significantly differ between the double (20.16%) and triple mutants (20.50%). Furthermore, our study found that there were no significant differences in crystallinity among the wild-type, double-mutant, and triple-mutant strains. In terms of branching degree, we observed that the single mutant had the lowest level, while the wild type, double, and triple mutants showed hardly any difference, similar to the degree of crystallinity (**Figure 6E**). This indicates that *sbe2b* greatly influence the branching degree, while *sbe1* helps to restore the reduced branching degree caused by the mutation of *sbe1*. Bringing into account the previous studies on the impact of crystallinity on starch digestibility (Chung et al., 2006; Sajilata et al., 2006; Zhou et al., 2018), our findings indicate that the increased RS content by mutated *sbe2b* is related to increasing the degree of crystallinity.

## Conclusion

In brief, our study revealed that the triple mutant exhibited the highest amylose and RS content, followed by the double and single mutants, while the lowest was in the wild type. Additionally, it was observed that the over-expression of *Wx^a^* in the triple mutant significantly improved the seed setting rate and 1000-grain weight. These findings suggest that an increase in the number of mutated genes could further enhance the RS content. This provides valuable insights into improving RS content through gene-editing in starch biosynthesis-related genes. Furthermore, it offers valuable information for breeding rice varieties with higher RS content and favorable agronomic traits as soon as possible.

## Data Availability

The raw data supporting the conclusions of this article will be made available by the authors, without undue reservation.
