# Supplementary material for: Triple gene mutations boost amylose and resistant starch content in rice: insights from sbe2b/sbe1/OE-Wxa mutants
Source: Front Plant Sci. 2024 Aug 14;15:1452520. doi: 10.3389/fpls.2024.1452520 (PMC11350245; doi:10.3389/fpls.2024.1452520)
Supplement: Supplementary file 1 [file DataSheet1.pdf]

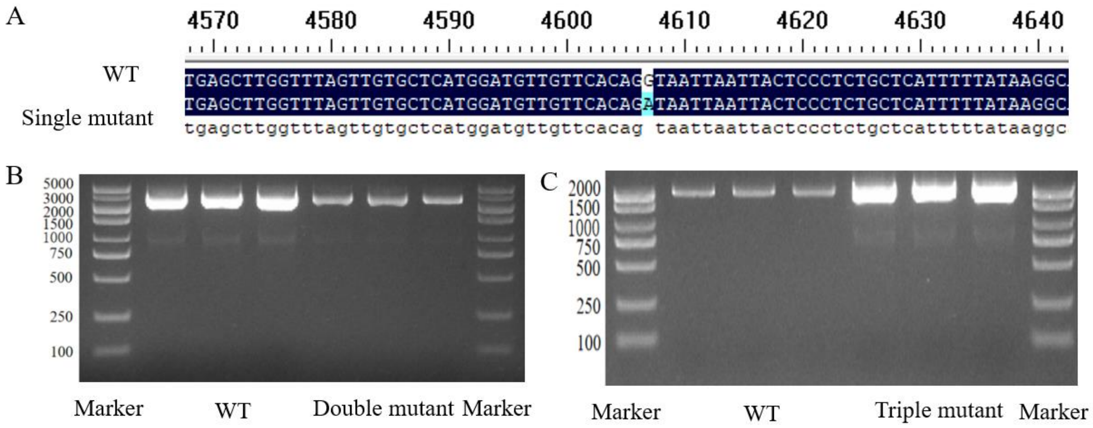

Fig.S1 Sequence of WT and single mutant and Gel bands of *sbe1* and *Wx*^{a}

Note: A, Sequence of *sbe2b* in *WT* and single mutant; B, Gel band of *sbe1* in WT and double mutant; C, Gel band of *Wx*^{a} in WT and triple mutant. Single mutant represents mutation of gene *sbe2b*, and located at the splice site between exon and intron 11, with a change from G to A; Double mutant represents mutation of genes *sbe2b* and RNAi-*sbe1*; Triple mutant represents mutation of genes *sbe2b*, RNAi-*sbe1* and over expressed gene *Wx*^{a}.
