# Supplementary material for: Triple gene mutations boost amylose and resistant starch content in rice: insights from sbe2b/sbe1/OE-Wxa mutants
Source: Front Plant Sci. 2024 Aug 14;15:1452520. doi: 10.3389/fpls.2024.1452520 (PMC11350245; doi:10.3389/fpls.2024.1452520)
Supplement: Supplementary file 2 [file Table1.docx]

Table S1 Primer identified for checking three mutants

| Primer name | Sequence (5'-3') |
| --- | --- |
| *BEIIb*-check-F | AGAAGATTTAAAGTCATTGA |
| *BEIIb*-check-R | CTAAGATAAACTCGAATGCT |
| *BEI*-check-F | AATGGAGATTTGAACGGCCG |
| *BEI*-check-R | AGAGTAACCACAAAACACCGA |
| *Wx^a^-*check-F | CAACATGTCGGCTCTCACCACGTC |
| *Wx^a^* -check-R | TACATCAAGGAGCAGCCACGTTCT |

Table S2 Primer of real-time PCR

| Gene | Gene ID | Forward (5'-3') | Reverse (5'-3') |
| --- | --- | --- | --- |
| *ACTIN* | Os03g0718100 | CTAAGCCAAGAGGAGCTGTTAT | ATAACAGATAGGCCGGTTGAAA |
| *Wx* | Os06g0133000 | AACGTGGCTGCTCCTTGAA | TTGGCAATAAGCCACACACA |
| *AGPS1* | Os09g0298200 | GTGCCACTTAAAGGCACCATT | CCCACATTTCAGACACGGTTT |
| *AGPS2b* | Os08g0345800 | AACAATCGAAGCGCGAGAAA | GCCTGTAGTTGGCACCCAGA |
| *SSI* | Os06g0160700 | GGGCCTTCATGGATCAACC | CCGCTTCAAGCATCCTCATC |
| *SSIIa* | Os06g0229800 | GCTTCCGGTTTGTGTGTTCA | CTTAATACTCCCTCAACTCCACCAT |
| *SSIIIa* | Os08g0191433 | GCCTGCCCTGGACTACATTG | GCAAACATATGTACACGGTTCTGG |
| *BEI* | Os06g0726400 | TGGCCATGGAAGAGTTGGC | CAGAAGCAACTGCTCCACC |
| *BEIIb* | Os02g0528200 | ATGCTAGAGTTTGACCGC | AGTGTGATGGATCCTGCC |
| *Pull* | Os04g0164900 | ACCTTTCTTCCATGCTGG | CAAAGGTCTGAAAGATGGG |
| *SUSY* | Os03g0401300 | AATGGTATCCTCCGCAAGTG | GGCTTGCATTTCCCTCATAA |
| *SUSY2* | Os06g0194900 | GCTGAAGGACAGGAACAAGC | CACCACAGACAACCACAAGG |
| *SUSY3* | Os07g0616800 | CATGTACCCCCTGCTCAACT | GTCAGCTGTAATGCCTGCAA |
| *ISA1* | Os08g0520900 | TGCTCAGCTACTCCTCCATCATC | AGGACCGCACAACTTCAACATA |
| *ISA2* | Os05g0393700 | TAGAGGTCCTCTTGGAGG | AATCAGCTTCTGAGTCACCG |
| *ISA3* | Os09g0469400 | ACAGCTTGAGACACTGGGTTGAG | GCATCAAGAGGACAACCATCTG |

Table S3 Agronomic traits of wild type and three mutants in different years planted

|  | Seed setting rate (%) | | | 1000-grain weight (g) | | |
| --- | --- | --- | --- | --- | --- | --- |
|  | 2021 | 2022 | 2023 | 2021 | 2022 | 2023 |
| Wild type | 90.91±2.93^d^ | 79.51±4.45^d^ | 89.25±3.29^d^ | 23.86±0.52^d^ | 24.18±0.33^d^ | 24.08±0.25^d^ |
| Single mutant | 85.46±4.34^c^ | 73.45±10.74^c^ | 84.12±7.37^c^ | 20.24±0.09^c^ | 19.80±0.27^c^ | 20.12±0.32^c^ |
| Double mutant | 56.81±2.39^a^ | 46.96±9.12^a^ | 49.67±5.76^a^ | 16.41±0.12^a^ | 15.18±0.41^a^ | 16.23±0.17^a^ |
| Triple mutant | 65.17±1.24^b^ | 62.67±8.93^b^ | 68.62±6.37^b^ | 20.29±0.24^b^ | 17.61±0.29^b^ | 19.86±0.12^b^ |

Note: Single mutant represents mutation of gene *sbe2b*; Double mutant represents mutation of genes *sbe2b* and RNAi-*sbe1*; Triple mutant represents mutation of genes *sbe2b*, RNAi-*sbe1* and over expressed gene *Wx^a^*. Values are expressed as the mean ± standard deviation (n = 3). Significant are compared among different sample in same year. Superscript letters represent significant differences at p < 0.05.

Table S4 Amylose and resistant starch content of wild type and three mutants in different year planted

|  | Apparent amylose content (%) | | | Resistant starch content (%) | | |
| --- | --- | --- | --- | --- | --- | --- |
|  | 2021 | 2022 | 2023 | 2021 | 2022 | 2023 |
| Wild type | 22.54±0.62^d^ | 22.19±0.92^d^ | 22.25±0.29^d^ | 0.62±0.10^d^ | 0.86±0.03^d^ | 0.75±0.05^d^ |
| Single mutant | 30.61±0.53^c^ | 30.57±0.74^c^ | 31.12±0.37^c^ | 1.68±0.07^c^ | 1.79±0.07^c^ | 2.41±0.02^c^ |
| Double mutant | 36.72±1.69^b^ | 35.87±0.48^b^ | 36.67±0.76^b^ | 3.02±0.12^b^ | 3.49±0.09^b^ | 3.13±0.07^b^ |
| Triple mutant | 43.01±0.97^a^ | 41.92±1.76^a^ | 43.62±1.37^a^ | 4.65±0.14^a^ | 4.63±0.22^a^ | 4.76±0.12^a^ |

Note: Single mutant represents mutation of gene *sbe2b*; Double mutant represents mutation of genes *sbe2b* and RNAi-*sbe1*; Triple mutant represents mutation of genes *sbe2b*, RNAi-*sbe1* and over expressed gene *Wx^a^*. Values are expressed as the mean ± standard deviation (n = 3). Significant are compared among different sample in same year. Superscript letters represent significant differences at p < 0.05.
